# The dipeptide conformations of all twenty amino acid types in the context of biosynthesis

**DOI:** 10.1186/s40064-015-1430-8

**Published:** 2015-11-04

**Authors:** Robert P. Bywater, Valera Veryazov

**Affiliations:** Magdalen College, High Street, Oxford, OX1 4AU England, UK; Francis Crick Institute, London, NW7 1AA England, UK; Department of Theoretical Chemistry, Lund University, POB 124, Lund, 22100 Sweden

## Abstract

There have been many studies of dipeptide structure at a high level of accuracy using quantum chemical methods. Such calculations are resource-consuming (in terms of memory, CPU and other computational imperatives) which is the reason why most previous studies were restricted to the two simplest amino-acid residue types, glycine and alanine. We improve on this by extending the scope of residue types to include all 20 naturally occurring residue types. Our results reveal differences in secondary structure preferences for the all residue types. There are in most cases very deep energy troughs corresponding either to the polyproline II (collagen) helix and the α-helix or both. The β-strand was not strongly favoured energetically although the extent of this depression in the energy surface is, while not “deeper” (energetically), has a wider extent than the other two types of secondary structure. There is currently great interest in the question of cotranslational folding, the extent to which the nascent polypeptide begins to fold prior to emerging from the ribosome exit tunnel. Accordingly, while most previous quantum studies of dipeptides were carried out in the (simulated) gas or aqueous phase, we wished to consider the first step in polypeptide biosynthesis on the ribosome where neither gas nor aqueous conditions apply. We used a dielectric constant that would be compatible with the water-poor macromolecular (ribosome) environment.

## Background

There are many reasons why there has been so much interest in calculating peptide conformations (Gould et al. 1994; Wu et al. 2010; Bellesia et al. 2010; Hovmoller et al. 2002; Bywater and Veryazov 2013; Carrascoza et al. 2014). These include the need to understand the preferred conformations of physiologically active peptides, the way peptides are incorporated into polypeptide and protein structures, and the conformation of *de novo* peptide formation in the ribosome. Most previous studies (Gould et al. 1994; Wu et al. 2010) were concerned with small peptides *per se*, in the gas or aqueous phase, while we addressed the latter question, that of peptide biosynthesis.

Throughout this and our previous work, and in keeping with the usage adopted by previous authors (Gould et al. 1994; Bywater and Veryazov 2013; Carrascoza et al. 2014), we study constructs that we refer to as primitive dipeptides with a *N*-acetyl-(XXX)(2)-*N*′-methylamine as a generic structure in which XXX represents the defining amino acid residue type for the particular dipeptide. In this context, *N*-acetyl is employed as a surrogate for the first amino acid residue in the dipeptide. Although not a true amino acid residue as such it is needed, together with the C-terminal amide group, to provide the correct electronic arrangement for a dipeptide and in order to block zwitterion formation. We chose to study all twenty members of the canonical set of amino acid types. A previous publication (Carrascoza et al. 2014) also reported studies of the entire set of amino acids, with somewhat different results, as discussed below.

As referred to in earlier papers (Bywater et al. 2001; Bellesia et al. 2010; Hovmoller et al. 2002; Bywater and Veryazov 2013; Carrascoza et al. 2014), different residue types have different propensities to adopt one or other of the regularly repeating polypeptide structures, α-helix, 3_10_ helix, polyproline II helix (here abbreviated as PP-helix) or β-strand (Bywater et al. 2001; Liljas et al. 2009). These preferences are however not absolute, they can vary according to context: both near neighbours and internal 3D contacts can affect the outcome. In many different areas of protein science it is of interest to know what are the energetic differences between these conformations. In the area we wish to investigate, that of the conformation adopted by newly synthesized peptides on the ribosome, it has previously been proposed (Lim and Spirin 1984, 1986) that the α-helix is the predominant structure. Our earlier results (Bywater and Veryazov 2013) support this prediction, but an alternative, the PP-helix, emerged as an equally likely and in some cases stronger contender. Furthermore, any extended α-helix would be vulnerable to disruption upon the appearance of a proline residue (Bywater et al. 2001). These reflections added to the importance of studying all twenty amino-acid types so as to see how these preferences are distributed throughout the entire set. It is important to note that while the α-helix and the β-strand are, either singly or in combination, by far the most predominant secondary structure types found in globular proteins (membrane proteins are either all-α-helix or all-β-strand), for fibrous proteins the converse is true, these are typically proline- and glycine-rich structures similar to the PP-helix which plays a prominent role. The protein biosynthesis machinery must be able to cater for both classes of protein.

## Methods

We constructed the starting structures for each of the many thousands of calculations in the same way as before (Bywater and Veryazov 2013) using the Yasara protein modelling program package (Krieger et al. 2002). A complete set of conformers was constructed for each set, whereby the C_i-1_–N_i_–CA_i_–C_i_ angles (the φ angle) were stepped through at intervals of 3° (120 steps) while for each φ rotamer the N_i_–CA_i_–C_i_–N_i+1_ angle (ψ) was stepped through 120 steps of 3°. This produced a total of 1681 structures for each amino acid type (41 for the special case of proline). In contrast to certain other studies (e.g. Carrascoza et al. 2014) there was no attempt to optimize these input structures. Instead a so-called rigid scan regime was imposed whereby, for each amio acid type, the rotameric state of the side chain was maintained while the φ,ψ angles were changed. This was considered essential in order to be able to make like-for-like comparisons for each amino acid type at these different backbone angles. If the side chain rotameric state for each different backbone geometry were allowed to relax, that would produce an energy minimized structure, but that would be a rather uninteresting object of study because it could not be compared with the thousands of other backbone geometries. Furthermore, the minimum side chain energy state may or may not be relevant at all. For all but the “smallest” side chain types there are multiple rotameric states that are accessible (Ponder and Richards 1987; Pupo and Moreno 2009). It would be impossible to cater for all of them.

For each of these conformers DFT calculations with B3LYP functional and ANO-L-VDZP basis set were performed using Molcas 7.8 (Aquilante et al. 2010). The PCM model was used to simulate solvation effects (Karlström et al. 2003; Pomelli and Tomasi 1997). As before (Bywater and Veryazov 2013) we selected a dielectric constant of 2.5 to reflect the water-poor environment of the peptidyltransferase site and the extremely slow tumbling rate of an object as large as a ribosome.

## Results

**Fig. 1:**
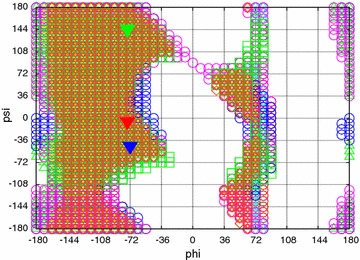
Generic φ,ψ map showing commonly populated areas. This figure is intended to be used as a template for labelling axes and determining values for the dihedral angles in Fig. 4. *Blue triangle* α-helix, *red triangle* 3_10_ helix, *green triangle* PP-helix

**Fig. 2:**
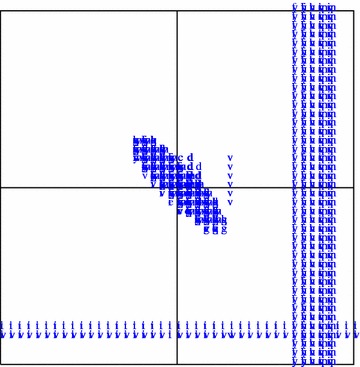
Generic φ,ψ map showing commonly forbidden areas. For this plot, “forbidden areas” is defined as those representing structures in which there is a close contact (“collision”) between atoms. The contact distance was set at 0.93 Å. These forbidden areas are generally less interesting than the “valleys” of the energy surface but they explain why certain residue types behave the way they do (in particular V, I and T). Residue types are shown in *lower-case single letter code*. This figure also explains the *black regions* in some of the members (especially V and D) of Figs. 3, 4, 5 and 6—the energy gradients are too steep to be properly rendered by the *graphics*

**Fig. 3:**
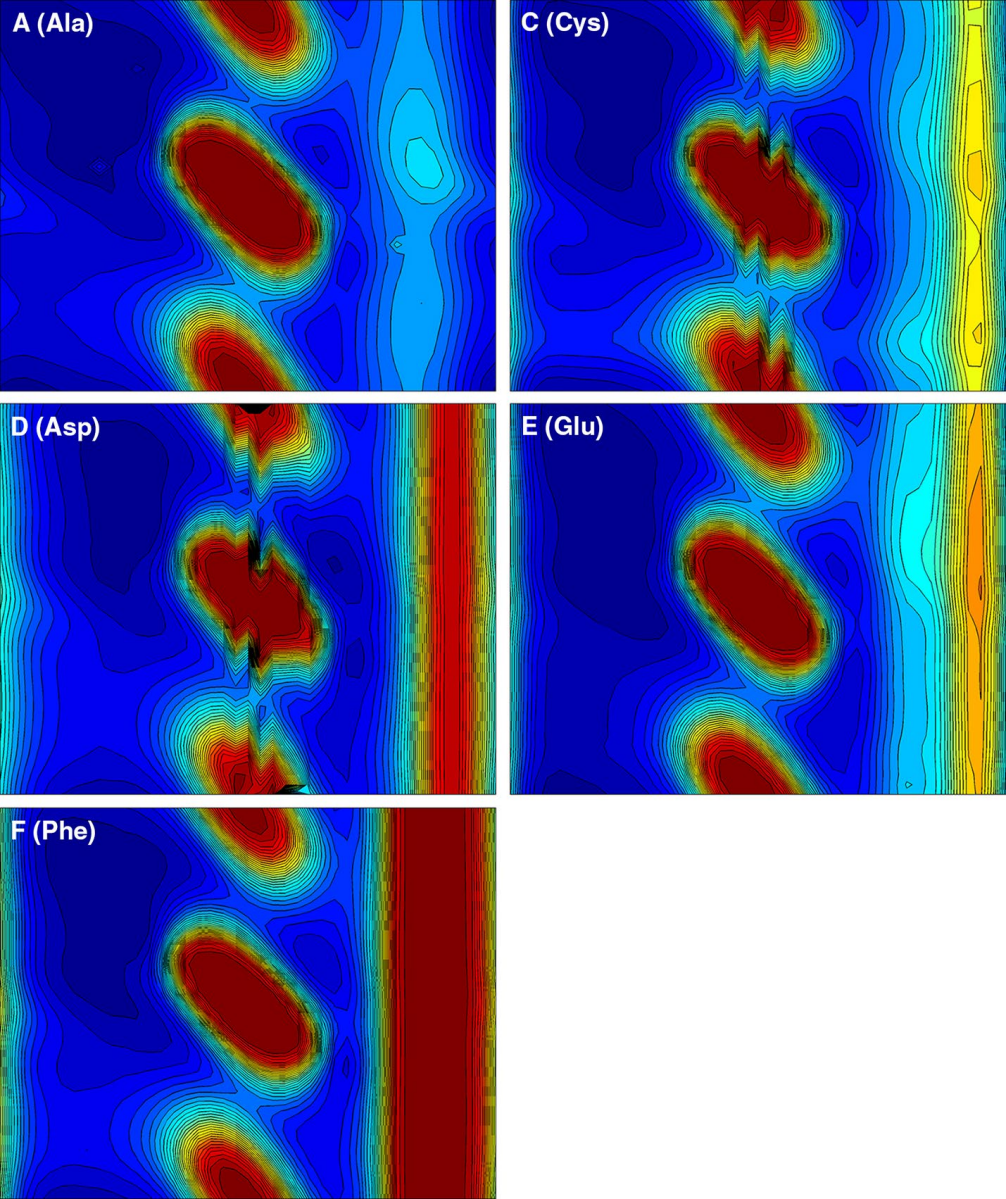
Φ,ψ energy surfaces for dipeptides. Φ,ψ energy surfaces for dipeptides with residue types are shown in this order: A (Ala), C (Cys), D (Asp), E (Glu), F (Phe). Note that only the −72° ≤ Φ ≤ −60° region is relevant for Pro because of its cyclic structure involving the –N–CA–CB–CG–CD– atoms which restricts rotation around the Φ dihedral bond. A diagram showing the chemical structure for Pro is provided in order to illustrate this. Some members of this set of dipeptides appear to show defects (*black colour*) in certain regions. This has been anticipated and explained above (caption to Fig. 2)

**Fig. 4:**
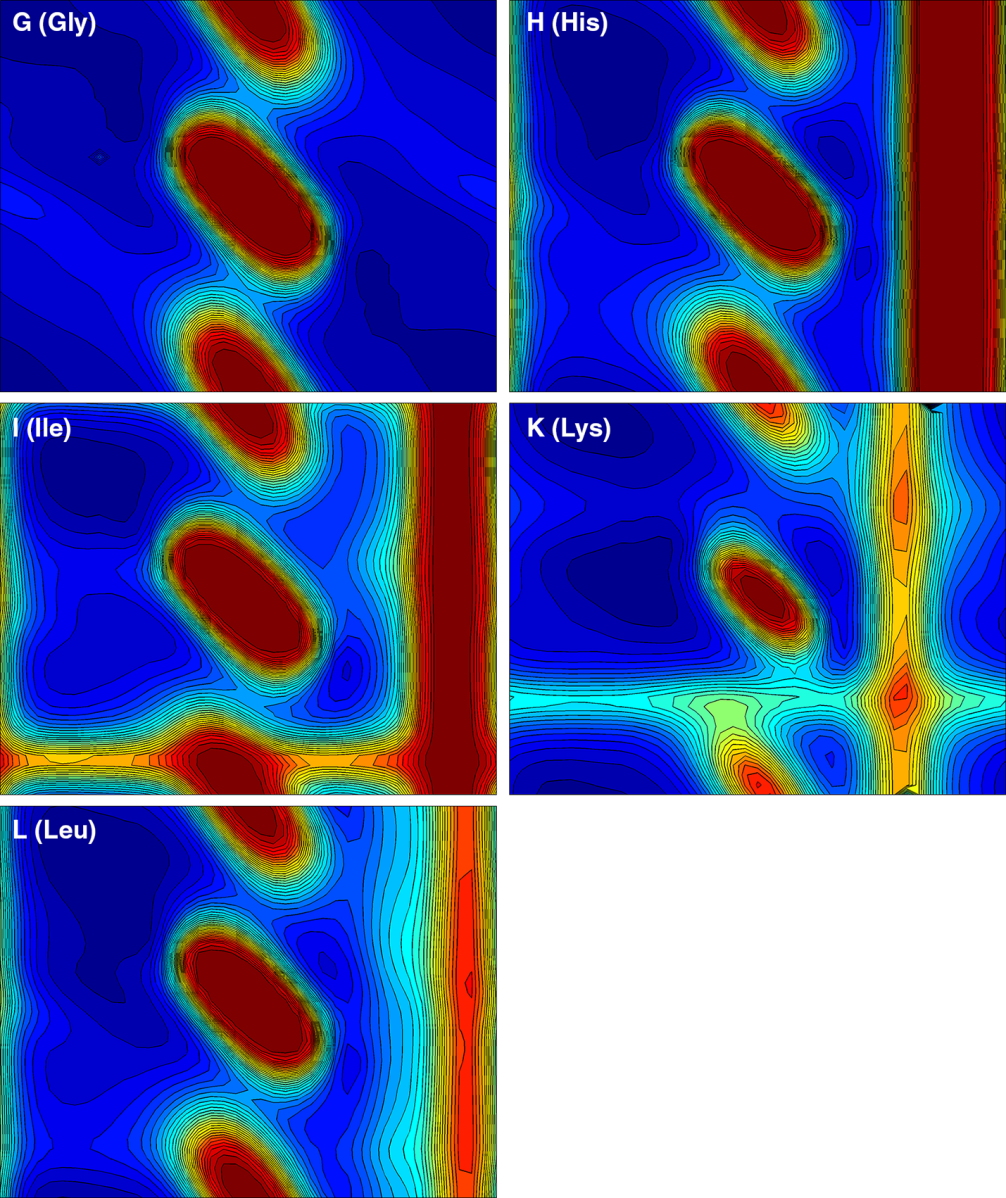
Φ,ψ energy surfaces for dipeptides. Φ,ψ energy surfaces for dipeptides with residue types are shown in this order: G (Gly), H (His), I (Ile), K (Lys), L (Leu). For details see the caption to Fig. 3

**Fig. 5:**
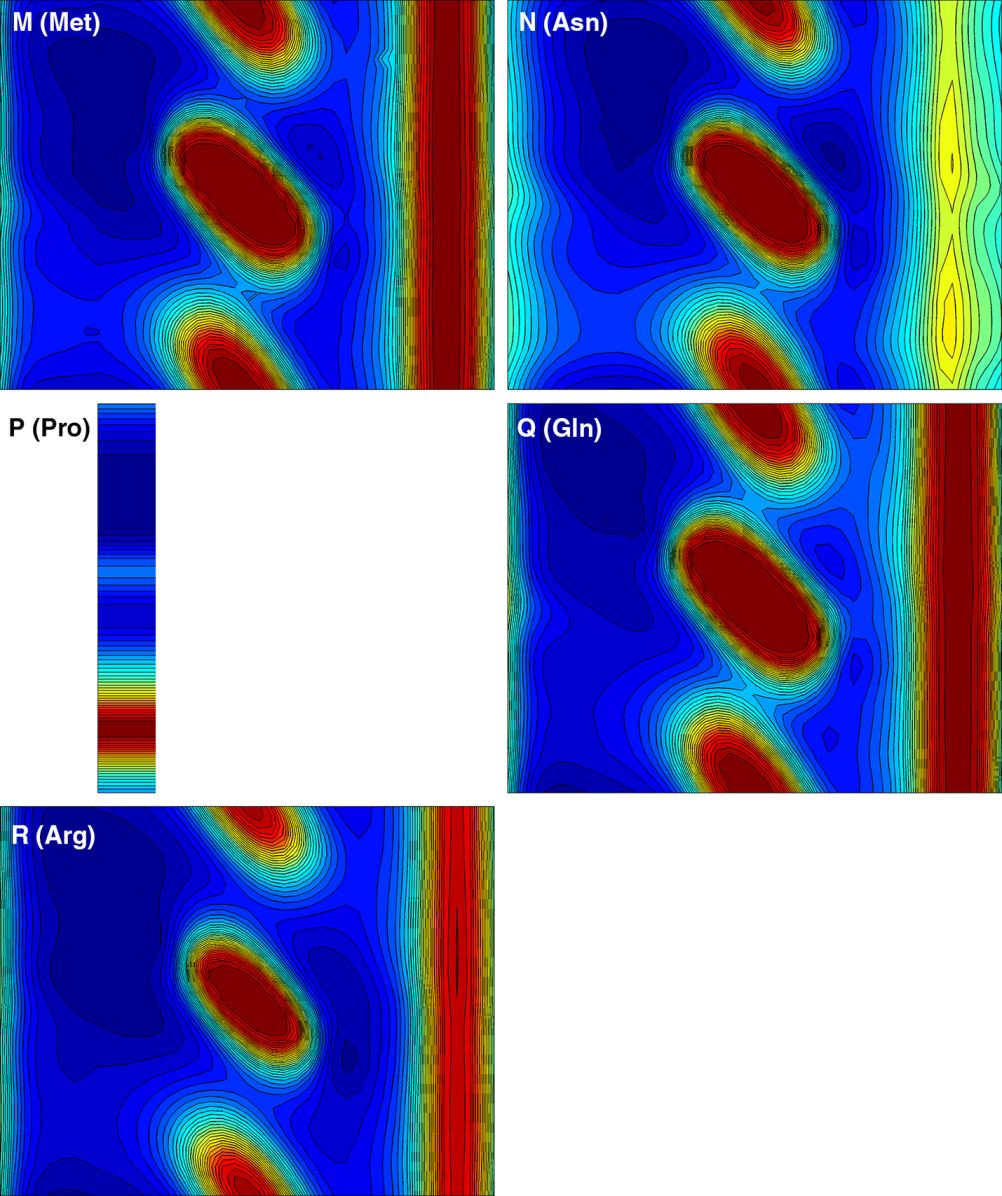
Φ,ψ energy surfaces for dipeptides. Φ,ψ energy surfaces for dipeptides with residue types are shown in this order: M (Met), N (Asn), P (Pro), Q (Gln), R (Arg). For P (Pro) the cyclic structure of sidechain locks the torsion angle. For details see the caption to Fig. 3

**Fig. 6:**
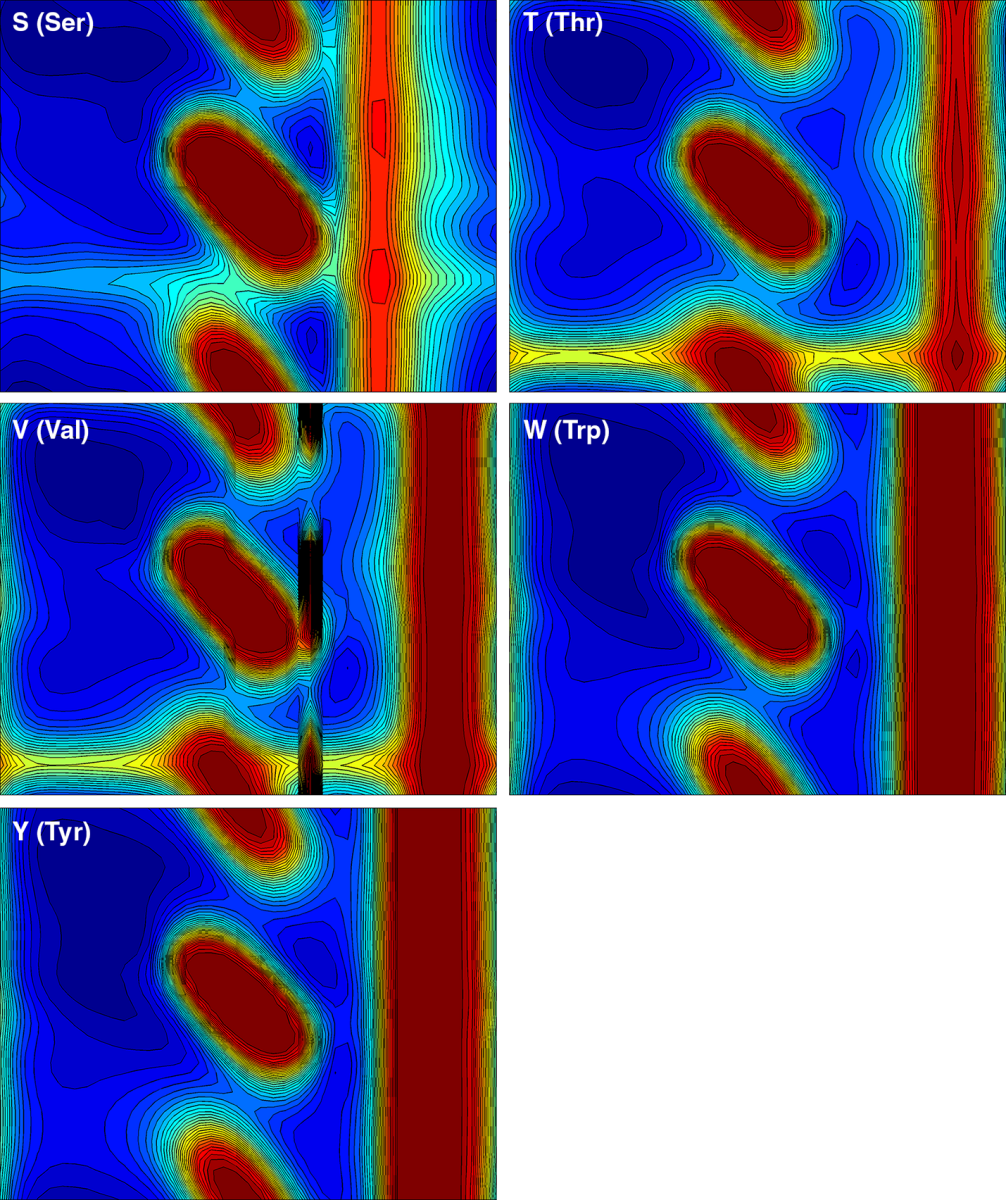
Φ,ψ energy surfaces for dipeptides. Φ,ψ energy surfaces for dipeptides with residue types are shown in this order: S (Ser), T (Thr), V (Val), W (Trp), Y (Tyr). For details see the caption to Fig. 3

The results of our calculations for the 20 residue types are presented in the form of Ramachandran-style energy surface plots for each residue type and a table that summarizes the salient features of each of these plots. Some necessary auxiliary information is required as a preliminary, this is provided in the form of the first two figures. Figure 1 is a graphical overview Ramachandran plot showing the φ,ψ positions of the 50 lowest energy conformers for all amino acid types except G and P. The location of the three classical secondary structure types α-helix, 3_10_-helix and PP-helix are shown by colored triangles (see caption to Fig. 1). Figure 2 focuses on the forbidden regions. This is intended to highlight some characteristics of certain residue types (in particular I, V, T and D) and to explain some features that turn up in Figs. 3, 4, 5 and 6. Further details are given in the caption to the figure. The grid and axis markings of Fig. 1 can be used for scaling the 20 plots in Figs. 3, 4, 5 and 6 [the β-strand region (not marked in the figure) covers a very wide range 100° < φ < 180°, 90° < ψ < 180°]. We note however that the large central forbidden region in our plots is almost absent in those of Carrascoza et al. 2014. The full set of results are displayed in Figs. 3, 4, 5 and 6, Ramachandran-style plots showing the φ,ψ distributions separately for each residue type (20 plots) with energy contours shown. There are 1680 data points for each plot (except P) and in order to give a better representation of this data a scaling factor ${\rm{tanh}} \sqrt {(e^2 - e^2_{\rm{max}})/10}$ was applied. For residue type P, only the region −72° < Φ < −60° is shown (40 data points). Because of the cyclic structure of its side chain involving the atoms which form the φ torsion angle (C′-N-CA-C), there are essentially no structures outside that range. As stated above, the key findings from a perusal of these plots is provided in Table 1 which describes the topography of the energy surface in φ,ψ space and provides remarks concerning the secondary structure preferences for each residue type.

**Table 1:** Description of the topography of the energy surface in φ, ψ space with remarks concerning the secondary structure preferences for each residue type

Consensus ranges for secondary structure types	α-helix	3_10_ helix	PP-helix	β-strand
	−72° < φ < − 60° −45° < ψ = − 39°	−75° < φ < − 74° −5° < ψ < − 4°	−72° < φ < − 76° –145° < ψ < −144°	100° < φ < 180° 90° < ψ < 180°

Residue type	Remarks
A (Ala)	Ramachandran plot shows a classical pattern with −30° < φ < 30° forbidden zone. The β-strand and PP-helix are well populated while α-helix is not strongly favoured. The right-hand region which is fully accessible to glycine (see G) is weakly accessible to A compared to other (non-G) amino acid types
C (Cys)	Very similar to A, but an additional forbidden zone shows up on the far right (φ ≈ 152°). Like A, β-strand and PP-helix are well populated while α-helix is not strongly favoured
D (Asp)	Very similar to C the right-hand forbidden zone broader now due to the bulk of the side chain carboxylate moiety. PP-helix and 3_10_ helix preferring rather than the “more famous” α-helix and β-strand.
E (Glu)	Very much like D except now the α-helix comes more into prominence (polyglutamate or glutamate-rich peptides are known to favour the α-helix). E also has a side chain carboxylate moiety but it is displaced further away from the peptide chain by an additional methylene, so the forbidden band is narrower than that for D
F (Phe)	Even distribution amongst all secondary structure types but very reminiscent of E. F is likewise known to favour the α-helix. Strongly forbidden 120° < φ < 152° region due to bulky aromatic side chain
G (Gly)	Essentially symmetrical distribution about the universal −30° < φ < 30° barrier. β-strand and PP-helix dominate. G can occur within α-helices but this residue type uniquely does not favour the standard right-handed geometry over a left-handed one, both isomers are equally possible [for A that would be an extremely rare event (but not unknown)]. Because of its rotational flexibility G is an important turn motif
H (His)	Similar to F but now with α-helix not strongly favoured. But like F has a very prominent 120° < φ < 152° restricted region
I (Ile)	This residue type offers a major difference to most of the others. A “new” restricted region −120° < ψ < −140° appears, indicative of significant steric clashes due to β-branching [NB. In this context “β” means that the branching occurs at the CB atom, as with T and V (*qv*)]. As for the allowed regions, the polyproline region φ = −72°, ψ = 144° is evident while the α-helix region φ = −72°, ψ = −45°, is considerably eroded (this was already reported in Bywater and Veryazov 2013 and similar findings were reported recently in Ilawe et al. 2015). The β-strand region shows up very prominently. This is as expected from experimental data (Bellesia et al. 2010; Hovmoller et al. 2002). We put the ranking for this residue type in the order polyproline > α-helix ∼ β-strand ≫ 3_10_ helix
K (Lys)	Resembles E in many ways but now there is a clear gap between the β-strand/PP-helix region and the (favoured) α-helix region. The 3_10_ helix region seems to be excluded (this may have significance for protein folding since 3_10_ helices can play a role in this process). One very striking feature, with it shares only with S (see below) is that the large barrier to rotation of the φ angle (usually 120° < φ < 152°) is shifted to φ ≈ 108°
L (Leu)	Similar to C and distinctly different from its position isomer I (*qv*). The absence of the −120° < ψ < −140° steric clash accounts for the different secondary structure propensities between L and I. In particular, L is amenable to the α-helix geometry while I is not. L does not seem to favour the PP-helix, and the β-strand region and α-helix regions are discontiguous
M (Met)	M is similar to F. For example, it is “α-helix friendly”. The 120° < φ < 152° forbidden zone shows up prominently. This restricted zone is due to the bulkiness of the side chain [in the interior of proteins, M often “behaves like” F (and W, Y) due partly to this bulkiness but also due to quantum chemical considerations concerning the somewhat similar behaviour of d-orbitals compared with the π-orbitals of aromatic side chains. These allow opportunities for orbital overlap which confers directionality]
N (Asn)	N is similar to D favouring the PP-helix and 3_10_ helix rather than the more famous α-helix and β-strand. But, while D is not regarded as being “α-helix preferring” exactly, N has a an even greater aversion and can be considered “α-helix forbidding”. The only difference between N and D is the amido-terminal group of the side chain instead of a carboxylate
P (Pro)	The plot for P is necessarily restricted to a very narrow strip in the Φ dimension due to its cyclic structure. As expected, P favours PP-helix almost by definition. But α-helix is a good runner-up. The notion that P is “helix-breaking” needs to be revised. P can sit at the beginning of an α-helix and even in the middle of such a helix (Bywater et al. 2001), although there will be disruptions at the (i − 3)rd residue (so-called “kinks”). But for P (where only the −60° < Φ < −72° region is relevant for this residue type (see Fig. 5) one can clearly discern the order of preference as PP-helix > α-helix ⋙ anything else
Q (Gln)	One might expect this to be similar to E. But it isn’t. Compared to E there is almost no preference for α-helix. This has to be a most significant result. How can amidation of a side chain make such a difference? But it mirrors exactly the difference between N and D
R (Arg)	One might expect R to resemble K. But, unlike K there is no divide between the α-helix and the PP-helix/β-strand region. These areas are effectively contiguous and 3_10_ helices would be accessible
S (Ser)	One might expect S to be similar to C (*qv*) but it isn’t. There is a much more pronounced 36° < φ < 136° zone and α-helix propensity is greatly diminished. The explanation probably has to do with intraresidue hydrogen-bonding. As noted with K (*q.v.*) the φ rotation barrier is shifted, this time to ≈96°
T (Thr)	Similar to S in the 36° < φ < 136° zone and almost exactly like I (and V) (*qv*) in the −120° < ψ < −140° region, indicative of significant β-branching causing steric clashes. Enhanced α-helix propensity compared with S
V (Val)	As with I and T: the −120° < ψ < −140° region highly restricted. Greatly diminished α- and 3_10_ helix propensity, β-strand dominant. An interesting incursion into the −80° < ψ < −120° region not really seen with any other residue types
W (Trp)	Very similar to H (and Y, F, M) due to bulky side chain
Y (Tyr)	Very similar to H (and W, F, M) due to bulky side chain

## Discussion

Our previous results, for residue types G, A, I and L provided support for established ideas (Lim and Spirin 1984; Lim and Spirin 1986) that the α-helix is a “default” conformation for the *de novo* generation of polypeptides on the ribosome but also demonstrated a clear alternative or rival. The PP-helix was given comparable, if not in some cases, greater prominence. We see further examples of that here, in the now extended repertoire of residue types. This is important because there is for any species only a single class of ribosome which has to cater for both globular (requiring α-helix and/or β-strand) and fibrous (strongly PP-helix preferring) proteins. Recent DFT studies on a restricted set of GXG model peptides (Ilawe et al. 2005) confirm the prominence of the PP-helix, while also finding a preference for β-strand. The latter is understandable since these authors were focusing on the X = I/V/L and the first two of these residue types are known (and shown here) to be β-strand preferring. All of these “preferential” states (α-helix, β-strand, PP-helix) must be regarded as at least potentially accessible for most amino acid types. I and V do turn up in α-helices, albeit less frequently than in β-strands. Note should be taken of the fact that while α- and PP-helix occupy a relatively small area of φ,ψ space these two structural types are characterised by very deep depressions which renders them enthalpically favored. The β-strand in contrast covers a wide area (alternatively: there is greater tolerance to distortions) although the depression is not as deep. Located between the α-helix, β-strand zones is a region that corresponds to the 2.2_7_ ribbon structure. This was discussed at length in Carrascoza et al. 2014 and indeed, our results do not rule out that some of the amino acid types might dwell in that region. But it is not normally found in proteins and it is an unlikely contender as part of a biosynthesis process. Concerning the apparent propensities for an α-helix geometry, this has to be viewed in the light of the fact that we are considering dipeptides and a true α-helix will not actually form in stretches shorter than 4 residues, in which the first of the hydrogen bonds that stabilize the helix can be established. So this suggests that there is something that intrinsically favours this helix regardless of the assistance provided by hydrogen bonds. The answer almost certainly resides in the need to “remove bumps”, i.e., steric repulsions between the atoms at certain key side chain torsion angles. Similar remarks might be made about the β-strand. There is a very wide range of backbone torsion angles available to this geometry. Also in this case there are no stabilising hydrogen bonds, but in proteins, β-strands are always incorporated into β-sheets, held together by hydrogen bonds. These β-sheets exhibit, as mentioned above, a very large variety of “shapes” and contortions which are allowed because of the very wide range of torsion angles accessible to the constituent β-strands. Lastly, mention should be made of 3_10_ helices. There are clear hints of distinct differences in their prevalence between different amino acid residue types and this can have repercussions for how protein folding takes place. Now that we have energy calculations for the entire set of 20 residue types this makes it easier to survey the whole family and see what patterns of secondary structure preferences might emerge.

The results presented here can be used by protein chemists as a guide to what the most likely secondary structure propensities are for each of the amino acid types. But certain caveats need to be issued. Firstly, the structures studied are not in the strict chemical sense “correct” structures for the dipeptides in gas phase or solution. This is anyway not an endeavor of compelling interest. Here, we have attempted to mimic an environment that the incipient polypeptide chain might encounter in the interstices of the ribosome, or indeed anywhere inside the cell which is known to be very “crowded”, but we can only do that with a very primitive solvation model. We do not know what the neighboring residues in contact with the newly synthesized peptide are and what the precise geometric arrangement is. We only allow the two backbone angles φ and ψ to change, Given the uncertainties about the environment, it does not make sense to allow all other angles to relax and to conduct energy minimizations of these structures. We think that by conducting things in the way we have has at least thrown some light on to the question of how each residue type behaves in comparison with the others, and some information concerning secondary structure propensities is provided. Obtaining structural information about longer peptides is of course also of great interest, but different methodologies are needed for that, molecular dynamics rather than quantum chemical methods, and recent work (Nilsson et al. 2015) reports the results of such cotranslational folding studies. These data do not in any way contradict our results, quite the converse, but the example given was of a small protein with a tendency to form α-helical structure. It would be interesting to see if any attempt is made to detect cotranslational folding of a fibrous protein, in which case the collagen PP helix would come into play.

## Conclusions

There has been much interest in determining the structure of dipeptides. Usually these efforts have been restricted to the case of primitive dipeptides where the central residue type is glycine or alanine, and no account was made of the effect of solvent. Gas-phase conditions were assumed. Our previous work extended this coverage of the residue type repertoire to two further cases, that of leucine and its position isomer isoleucine. Simulated solvent conditions corresponding approximately to the water-poor environment and large particle size of a ribosome (or elsewhere in the crowded interstices of the cell) were applied. Already at that stage, major differences were seen between the four residue types, particularly between the two isomers. This encouraged further research into the entire set of 20 standard residue types. We have produced a compendium that protein chemists can use as a guide to the most likely secondary structure propensities for each of the amino acid residue types. Most amino acid residue types can access all three of the major secondary structures α-helix, β-strand, PP-helix but there are individual preferences which were known from experimental and bioinformatics studies. Our plots map out these preferences. In reference to ribosomes we recall that the same ribosomes have to cater for all 20 amino acid types but also enable both globular and fibrous proteins to be formed within and emerge from the peptide synthesis tunnel. We have not considered cotranslational folding as such, but our work should be helpful as a starting point for such studies.
